# Providing a model for the development of sports tours in the tourism industry

**DOI:** 10.1371/journal.pone.0285457

**Published:** 2023-05-25

**Authors:** Mahdi Gharibzadeh, Ali Mohammad Safania, Seyed Salahedin Naghshbandi, Abolfazl Farahani

**Affiliations:** 1 Science and Research Branch, Islamic Azad University, Tehran, Iran; 2 Payame Noor University, Tehran, Iran; University of Kurdistan Hewler, IRAQ

## Abstract

Sports tours are a key category of tourism with special interest whose evolution and development are influenced by a variety of limiters and facilitators. Research literature recommends exploring these factors. Therefore, our goal was to present a grounded theory about the development of sports tours in Iran’s tourism industry. For this purpose, we considered Glaser’s approach and conducted 15 interviews with experts in the field of tourism and sports tours who worked under the supervision of the Ministry of Tourism and Cultural Heritage of Iran. The data were analyzed using the Glaser’s approach principles. The developed theory from this study showed that the development of sports tours is an structure that stops moving under the influence of inhibitors such as financial, political, security, structural, and organizational issues, and travels the path of perfection and progress through facilitators such as natural attractions, the role of the media, the role of tour guides, the quality of services, information technology, support, culture, and training, and the human force. Based on this result, it can be said that the development and evolution of sports tours in Iran are influenced by various determinants, and it is important to remove the limiting factors and strengthen the factors that play a facilitating role in the development of sports tours.

## 1. Introduction

Among the service industries, the tourism industry is one of the leading and large sectors at the international level [1]. This industry is not only the largest but also one of the fastest-growing sectors of the world economy, whose growth rate is almost two times higher than the growth rate of other economic sectors. In terms of key indicators, including investment efficiency, tourism is comparable to the oil industry, because tourism is about 10% of the world’s total products; it accounts for 30% of global service exports, 7% of global investment, 10% of jobs and 5% of total tax revenue [2]. Therefore, nowadays most of the developed countries, knowing the significance of this industry, take the best advantage of tourism, while the less developed countries experience the opposite of this, despite the urgent need for employment and income generation [3]. The reason why developed countries are more successful in this area than less developed ones is that these nations have planned and implemented policies and macro-strategic plans for this area. Also, they have made the best use of the capacity of tourism, which has various types [4].

It is worth mentioning that there are various types of tourism, and sports tourism is one of them which has grown the most among the different sectors of this industry, because people used to travel to participate in sports festivals since ancient times [5]. Nowadays sports tourism has become a fashion. According to the statistics of the World Tourism Organization, in 2014, sports tourism accounted for 15% of the tourism market, and it is expected to increase to more than 30% by 2020 [6]. For this reason, sports tourism has become a crucial part of the tourism industry in many countries today [7].

One of the important parts of sports tourism is sports tours, which are often carried out in the form of recreational activities [8]. Also, sports tours are defined as one of the sports tourism sectors which include various activities such as professional sports games tours, sports study tours, sports adventure tours, event, places and facilities tours, educational tours, cycling tours, hiking tours, climbing tours, caving tours, etc. [9]. While considering the activities of this sector of tourism as important, Kirillova believes that the daily expenses of sports tour tourists are higher than other types of tourists. Accordingly, it can be viewed from the perspective of a tourism market with high potential [10]. It is necessary to consider certain plans and programs for their development and progress, as in other areas of sports tourism, because tourism is one of the most important sectors of the tourism industry with a production of more than 250 billion dollars and annual increase of more than 9% has the highest growth rate among various tourism sectors [11].

In the last decade, there has been an increasing growth in academic studies of sports tourism and tourism with special interests on how to develop it, and researchers have addressed this issue from both qualitative [12–14] and a quantitative [15–19] perspectives. However, surprisingly, there is no consistent and substantiated research on sports tours as one of the pillars of tourism with special interests and sports tourism. This is despite the fact that sports tour is not considered as a new type of sports tourism, but still there is not enough knowledge about this type of tourism. Also, it is not clear what outputs it is subject to as a structure, or what are its facilitators and limiters. Another issue is that in Iran, few tourism companies enter the field of sports tours and take advantage of the capacities of this field. Because there is no sufficient understanding of the capacities and potentials of this type of sports tourism, there is no sufficient understanding of the problems and challenges associated with this type of tourism in Iran, and the mentioned companies do not have enough information about how they can bring this type of tourism to perfection. Due to this lack of knowledge authorities failed to properly prioritize sports tours in Iran’s main tourist development policies. Despite the fact that sports tours are a particular kind of social phenomena that requires an appropriate and sufficient foundation in order to express in society, this is the case.

Acknowledging the gaps in the research, we raise our research questions:

- What are the accelerators or facilitators of sports tours in Iran?
- What are the inhibitors or limiters of sports tours in Iran?

The purpose of this study is to create a conceptual model to explain the facilitators and limiters of sports tours in Iran. In this study, the inductive qualitative approach, grounded theory was used [20–22]. The grounded theory is an "inductive" method with a completely "qualitative" approach and an "exploratory" research method. It provides the possibility for researchers to, in cases where it is not possible to formulate a hypothesis, instead of using predefined theories, to personally develop a new theory. However, this new theory is not based on the personal idea of the researcher but on the data provided by the environment and real conditions. Also, grounded theory has been used to study processes and explain why and how they happen, which is consistent with the purpose of our study [23].

A theoretical foundation for the development of sports tours is anticipated from the findings of this study. It serves as a reference for tourism companies as well since it gives them new perspectives on how to enhance and engage in tourism with special interests, especially sports tours.

The structure of this study is as follows. In the second part, we review the texts related to tourism with special interests, sports tourism, development of sports tourism, and sports tours. Then, in the third part, we introduce the methodology of this research and its method. The research findings are discussed in the fourth section and the results in the fifth section. The last part shows the limitations of this research and suggests future research directions.

## 2. Literature review

### 2.1. Tourism with special interests

Tourism is an ancient phenomenon that has been a part of human society for a very long time. It has changed and evolved over time as a result of economic, sociocultural, and technological factors. The tourist market and demand have changed significantly in recent years, particularly in the 1980s and 1990s, as a result of the fast urbanization, communication, and technological advancements [24]. These changes have been accompanied by the formation of new destinations and experiences, increasing the diversity of tourism products and tourism with special interests, which is one of the examples of the postmodern world [25]. Tourism with special interests refers to specialized tourism including groups or people who want to develop special interests by visiting sites and places related to a specific topic [26].

Special interest tourism includes enjoying activities in contact with nature, the beauty of a protected natural area, enjoying watching animals in their natural habitat, as well as exploring, discovering and learning, overcoming obstacles, and feeling the joy of overcoming them [27]. In this kind of tourism, tourists are often looking for exciting stimuli and gaining experiences full of complexity and excitement, along with special enthusiasts. A suitable market has good potential for creating employment and income [24]. It should be noted that special interest tourism has introduced a new definition of tourism to the modern world, which is fundamentally different from the past types and pursues goals beyond the common types, and of course, it is of interest to certain people [28].

### 2.2. Sport tourism

One of the types of special interest tourism is sports tourism [29]. Sports tourism refers to trips that involve watching a sports match or participating in such matches and includes three parts of sports event tourism, active sports tourism, and nostalgia sports tourism [30]. Sports event tourism includes major sporting events such as the Olympic Games or the World Cup football matches [31]. People who travel to do a sport and actually participate in a sports event are active sports tourism tourists. These collaborative events include a wide variety of sports and various forms of tournaments and events [32]. Also, nostalgic sports tourism refers to trips that include visiting sports tourism attractions [32]. Finally, sports tourism is one of the types of tourism, whose scope of activities includes 5 sections: Sports events, Sports events, Sports tours, Sports resorts, and Sports Cruises, and each section has different sub activities, and it is necessary for the relevant policymakers to pay enough attention to these activities in determining the policies for the promotion of sports tourism [9].

### 2.3. Development of sports tourism

The development of sports tourism, due to improving social welfare, improving the quality of life, improving the national economy, helping to increase the social, environmental, and cultural aspects of the host society, and many other desirable results and achievements, have always been the attention of the relevant stakeholders during the past decades [33]. Although for many developing countries and regions, it is a suitable option for development, however, it also has different costs and environmental effects and dependencies. Therefore, decision-making about how to develop tourism should be done carefully [34]. Regarding the development of tourism, various theories have been proposed, including the modernization theory, dependency theory, neoliberalism, and alternative development. Awang et al. [35] believe that development is progressive, as experienced in many countries. Development theory provides a suitable conceptual framework for explaining tourism development processes. This shows that these processes are not simply the result of unilateral actions. Instead, the nature of tourism development is highly debated, which is susceptible for being affected by the surrounding environment, which is invoked by factors such as politics, economy, culture, and environment among others. Such processes are further complicated by the fact that the tourism industry is made up of various interrelated areas and therefore has a wider scope and depth.

### 2.4. Sports tours

According to the definition of the World Tourism Organization, a tour refers to a predetermined trip that is prepared by tourism agencies and provided to tourists. Also, a tour in the tourism industry refers to a set of elements that are correlative to each other. These elements include transportation and movement, accommodation, food, attractions, executive factors, shopping, etc. [36]. There are many different types of tours in the tourism industry, some of which contribute the most to this field. One of these tours, which is also emphasized in the present study, is sports and adventure tours. Sports tours are defined as a set of travel components including accommodation, transportation, food, sightseeing, etc. which are provided by travel agencies or tour operators for a person or people from the group of sports tourists in the form of a package [37].

## 3. Materials and methods

### 3.1. Grounded theory

In this research, we choose grounded theory as a research method. Grounded theory methodology provides a tried-and-true set of procedures for building theory from data, which have been shown to be culturally sensitive and applicable to individuals as well as larger organizations and communities [21]. Additionally, the grounded theory includes systematic yet flexible guidelines for collecting and analyzing qualitative data to build theories from the data itself [22]. There are three types of grounded theory: traditional [20], evolved [21], and constructivist [22]. These grounded theory methods have different ontological and epistemological foundations [38], and are neither homogeneous nor interchangeable [39]. Because these three grounded theory methods are based on different research philosophies, none is superior to the others. The basis of our study lies in the evolved theory as described by Glaser.

### 3.2. Sampling and study participants

Sampling in grounded theory begins with the recruitment of individuals who are assumed to be able to provide accurate insight into the study questions [21]. The researcher was sensitive to the concept that the intended participants should be experts in the field of sports tourism and sports tours. Then, targeted sampling was used to select experts. The participants in this study had worked professionally in the field of sports tourism for at least 5 years and were members of the Ministry of Cultural Heritage and Tourism, travel and tourism service companies, and startups in Iran’s tourism field. The reason why the participants were selected from different spectrums was to examine a spectrum of viewpoints regarding the development of sports tours. After interview, from the 8th to the 12th interview, we experienced the repetition of the interview information, however, to ensure data saturation, we conducted three more interviews (Table 1).

**Table 1:** Demographic characteristics of the participants.

Row	Position/area of activity	Experience (Years)	Education	Field of study	Age (years)
P1	The employee of the ministry of cultural heritage and tourism	5	Ph.D. student	Political geography	29
P2	Managing director of nature tourism company	26	Masters	Tourism management	49
P3	Chairman of the sports tourism committee of the ministry of cultural heritage and tourism	9	Masters	Sport Management	47
P4	Head of the codification office of the ministry of cultural heritage and tourism	20	Ph.D. student	Tourism management	45
P5	Head of the general supervision department of the ministry of cultural heritage and tourism	15	Ph.D.	Tourism management	49
P6	Editorial board of the quarterly journal of tourism research and sustainable development	21	Ph.D.	Geography and tourism planning	43
P7	Business consultant in tourism start-ups	18	Ph.D.	Business Management	40
P8	Managing director of travel and tourism services company	17	Ph.D.	Business in Tourism	44
P9	Managing director of travel and tourism services company	17	Masters	Ancient languages of Iran	42
P10	Business consultant in sports tourism startups	6	Masters	Sport Management	37
P11	Managing director of a non-governmental organization active in the field of sports tourism	8	Ph.D. student	Sport Management	31
P12	Sports tour guide	17	Masters	Tourism management	48
P13	Managing director of nature tourism company	11	Ph.D. student	Tourism management	45
P14	Sports tour guide	8	Masters	Business Management	35
P15	The employee of the sports tourism committee of the ministry of cultural heritage and tourism	13	Ph.D. student	Tourism management	37

In Table 1, the demographic characteristics of the participants are listed in terms of gender, education, and field of study.

### 3.3. Data collection

The participants were asked to take part in several in-depth interviews aimed at investigating how sports tourism is developed. We chose online data collection because the temporal and spatial flexibility offered by the Internet favors qualitative research. The online method allowed us to reach diverse and geographically dispersed populations as well as informants that were not easily accessible due to time-table issues [40]. In addition, virtual anonymity and higher private self-awareness stimulate the disclosure of personal information and deep feelings [41].

### 3.4. Validity and reliability

Few researchers rely on the concepts of reliability, internal and external validity, and reproducibility to legitimize their studies. In contrast to their counterparts, namely, qualitative researchers, they confirm reliability, validity, transferability, and confirmability [20, 42]. In this study, since the grounded theory is used, firstly, the four terms presented around qualitative studies are defined and then the related strategies are mentioned.

Reliability: Reliability in qualitative research is similar to reliability in quantitative research. Reliability means that the instruments used to collect data produce consistent results between data collection events. For example, one meter of metal tape is likely to be more reliable than one meter of cloth tape, because one meter of metal tape does not stretch as much as one meter of cloth. Reliability can be estimated statistically. In qualitative studies, the concept of statistical reliability is not used. Rather, qualitative studies must meet a standard of reliability. Reliability means that there is evidence of consistency in data collection, analysis, and reporting. It also means that any adjustments or changes in methodology, which can occur in qualitative studies, are documented and explained in a way that is accessible to the public. Inquiry audit and triangulation are the most common methods of establishing reliability [42, 43], which were cited in this research.

Validity: Internal validity in quantitative research verifies that the collected data is consistent with the research question. In other words, if the research question is about motivation, the collected data should be about motivation. Validity is a parallel concept for qualitative studies. Credibility means that the findings of the study are believable according to the data provided [43]. Validity is built using strategies such as prolonged engagement, continuous observation, peer discussion, negative case analysis, progressive mindset, member checking, triangulation, and reflection. If a qualitative study is conducted, there is no need to use all strategies related to validity. Instead, the best strategy or strategies should be chosen for the study [42, 43]. Therefore, in the present study, strategies of long-term interaction, continuous observation, peer discussion, member review, and triangulation were used.

Transferability: Transferability is related to the quantitative concept of external credit. External validity provides a measure of the generalizability of study findings based on a sample to the study population. Although the goal of qualitative research is not to generalize from a sample to a population, a qualitative study must have meaning beyond the immediate study sample. Determining the applicability of the findings of a qualitative study in other situations is mainly the responsibility of the person in the other situation. Regarding transferability, the researcher’s responsibility is to provide a sufficient description of the environment and assumptions of the study so that the reader can use the findings of the study in an informed manner [44]. It is worth mentioning that in this study transferability is supported by using deep descriptions and maximum diversity [43].

Verifiability: The goal of quantitative research is to be objective, to extract the researcher from the study as much as possible in such a way that the research findings are separated from any bias of the researcher. Qualitative research accepts the researcher’s mentality, but its methods must be based on procedures, analyzes and verifiable conclusions. In other words, confirmability requires that other knowledgeable researchers reach the same conclusions when examining the same qualitative data [42]. Guba and Lincoln [42] suggested verifiability audit as the main tool to create verifiability and this strategy was used in this study.

### 3.5. Data analysis

Data analysis started from the first day of data collection, which was done with the help of transcription, note-taking, coding, constant comparison and theoretical sorting, graphing, and integration. All interviews were conducted in the local language (Persian) and were transcribed as soon as they were completed. The primary author transcribed all recordings into Microsoft Word. Then the transcripts were checked and sent to the participants via email for review. This was done to ensure that the transcripts reflected the participant’s views. Each transcript was kept in its original form and was never translated into English because translating a large part of the data into another language risks losing the original meaning [45]. However, the generated themes or codes from the analysis were translated into English because they were shorter (in phrases or few words), easy to translate, and less likely to lose meaning.

To facilitate the process of data analysis, note-taking was done in three stages: "after each interview", "during transcription" and "during coding". They included reflections on the setting, how the participants expressed their feelings and responses, the quality of the interview, lessons learned, and strategies for future interviews. While transcribing, notes were focused on keywords, pattern questions and gaps to be filled. Note-taking during coding focused on the relationship between key ideas and themes that emerged during the analysis.

Coding is the core stage of analysis in grounded theory, which is the process of sorting and combining large amounts of data into concise and meaningful expressions with the development of categories and subcategories, and includes three stages: "open coding", "selective coding", and "theoretical coding". Based on the evolved (Glaser) approach, open coding continues with free coding of data until the effects of the appearance of the central category are charted, so that in the next stage, the selective coding stage, coding is guided based on the central category. In selective coding, coding is done selectively around and based on the central category. At this stage, the categories are listed and then different characteristics and groupings are done [46]. Finally, in theoretical coding, integration between categories is done by a communication pattern. This stage of coding allows the researcher to approach the thinking about the categories that may lead to a wide range of mental possibilities and to think analytically about the possible link between the categories. Theoretical codes are abstract models that combine categories and their characteristics towards a theory [47].

## 4. Results

**Fig 1:**
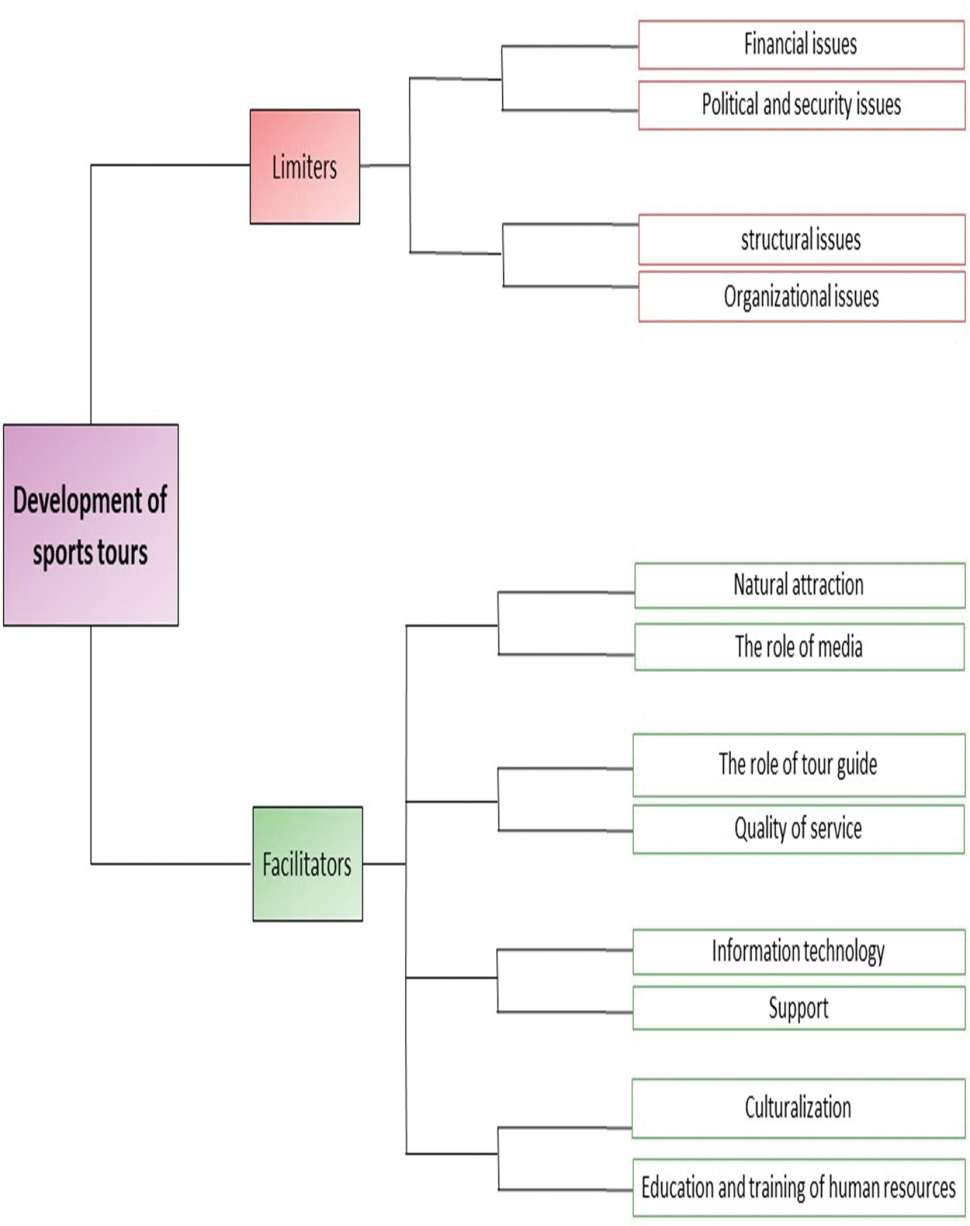
Model of sports tour development in the tourism industry. Financial, structural, organizational and political, and security issues are part of the limiters and the role of the media, natural attractions, the role of tour guide (leader), service quality, information technology, support, culture, and human resources education were one of the facilitators.

Fig 1 presents the model of sports tour development in the tourism industry, briefly. In general, our findings show that sports tours are prevented from developing by disturbing and limiting stimuli and move towards development through accelerating stimuli. Financial, structural, organizational and political, and security issues are part of the limiters and the role of the media, natural attractions, the role of tour guide (leader), service quality, information technology, support, culture, and human resources education were one of the facilitators. We will now discuss these two streams in more detail.

### 4.1. Limiters (inhibitors) of development

Limiters or inhibitors are those conditions and factors that widely limit the development of sports tours in Iran. These factors deeply and widely control the various dimensions of sports tours. The main category of restrictions consists of five subcategories of financial issues, structural issues, organizational issues, and political and security issues.

In Table 2, concepts and sub-categories extracted from the qualitative data related to development limiters are detailed.

**Table 2:** Concepts and sub-categories extracted from qualitative data related to development limiters.

Main Category	Subcategory	Concepts
**Limiters**	Financial issues	People’s purchasing power decline
		The high cost of tours
		The variable rate of tours
	Structural issues	Lack of interdepartmental/organizational coordination and integration
		Interference of duties of organizations related to tourism
		The multiplicity of decision-making centers
	Organizational issues	Lack of incentive policies for private sector participation
		Absence of comprehensive sports tourism development plan
		Non-specialized management of sports tourism
		Short-term policies in the field of tourism
	Political issues	Lack of belief in sports tourism
		Dependence on oil
		Diplomatic problems
		Negative propaganda against Iran
		Embargo
	security issues	Domestic incidents against international tourists
		International tensions

#### 4.1.1. Financial issues

This category means that the development of sports tours is limited by financial issues. Based on this category, people’s purchasing power has decreased compared to the past, and as long as people do not have adequate financial and livelihood capacity, they will not be able to meet their secondary needs as they are simultaneously involved in meeting their primary needs. Also, with the decrease in people’s purchasing power, the cost of sports tours in Iran has increased, and there is no specific basis for pricing sports tours, and the prices in this area are variable. In this context, we can refer to interviewee number 8’s quote:

"The development of sports tours is subject to financial stimuli, and it is not possible for sports tours to develop as long as financial stimuli are not positive." Unfortunately, in Iran, due to the economic conditions prevailing in the society, people do not have a high financial capacity, and rather than going out and having fun, they are thinking of leaving their daily lives."

#### 4.1.2. Structural issues

According to the participants, Iran’s tourism industry, especially sports tours, is associated with structural problems. These structural issues have made the development of sports tours a challenge. In all the interviews, one of the factors mentioned as structural issues was the lack of inter-departmental or organizational coordination and integration. This means that there is no necessary coordination between the different active departments and organizations in Iran’s tourism field, and these organizations operate in isolation and sometimes work in parallel with each other. One of the respondents, who was the head of the sports tourism committee of the Ministry of Cultural Heritage and Tourism, addresses this issue as follows: "There is no necessary coordination and coherence between the organizations under our supervision, each organization works for itself, and we, as the upstream organization in connection with sports tourism in the country, do not have the necessary desire and will to unite and coordinate these sectors, and this issue has caused serious damage to the development of the sports tourism industry. Interviewee No. 11 also says: "Furthermore, there is no coordination and coherence between the relevant organizations, it can be seen that the duties of the relevant organizations overlap, because there are many decision-making centers related to sports tourism in the country and these organizations are not specifically controlled by a specific trustee."

#### 4.1.3. Organizational issues

Another extracted subcategory is "organizational issues". The meaning of this category is that as a result of organizational issues and problems, the development of Iran’s tourism industry has been accompanied by challenges. Here, the lack of a comprehensive plan for the development of sports tourism in the country, non-specialized management of sports tourism and short-term policies in the mentioned field, and the lack of incentive policies for the participation of the private sector in the field of sports tourism are evident. From the point of view of the interviewee number 1: "The relevant issues, especially the lack of a comprehensive plan for the development of sports tourism in the country, is one of the problems that sports tourism in Iran suffers the most from, and since there is no comprehensive plan in this direction, most measures that are taken are temporary and do not bring macro and strategic results.

#### 4.1.4. Security issues

Security issues are very important as one of the limiters to the development of sports tourism. The three security reasons that stop the development of sports tourism in Iran are: negative propaganda against Iran, international tensions and internal incidents against international tourists. In this context, the following two quotes are noteworthy: "Unfortunately, due to the international tensions that the country is dealing with, tourists, especially foreign tourists, do not want to travel to Iran, and this issue has negatively associated the development of sports tourism with a serious challenge."

"During the years that I have worked as a tour guide, I have witnessed unfortunate incidents and events, and unfortunately, most of these incidents occurred when there were foreign tourists on my tour, this issue itself has caused the world, especially foreign tourists, to have a negative view of our country and not only have no desire to travel to our country but are also afraid of coming to our country."

#### 4.1.5. Political issues

Another limiter that has stopped the development of tourism in the country, and negatively contributed to the lack of development of the sports tourism industry is political issues. One of the most important issues that has been repeatedly mentioned in the interviews is the issue of sanctions. In this regard, the interviewees number 2, 4 and 11 simultaneously stated that "the embargo has paralyzed the sports tourism industry in the country, whether the embargoes are right or wrong is not important, it is important that through embargoes the sports tourism industry in the country is shrinking more every day than yesterday, and the current situation will not change until the sanctions are lifted." It should be noted that besides the sanctions, other political issues such as diplomatic problems, dependence on oil, lack of trust in tourism by the authority and negative propaganda against Iran are evident.

### 4.2. Facilitators (accelerators) of development

Facilitators or accelerators are those conditions and factors that greatly facilitate the development of sports tours in Iran. These factors are among the key drivers of the development of the sports tourism industry and positively contribute to the progress and improvement of sports tours. The main facilitation category consists of eight subcategories: the role of the media, natural attractions, the role of the tour guide (leader), service quality, information technology, support, cultural development, and training and education of human resources.

In Table 3, concepts and sub-categories extracted from qualitative data related to development accelerators are detailed.

**Table 3:** Concepts and sub-categories extracted from qualitative data related to development accelerators.

Main category	Subcategory	Concepts
The Facilitators	The role of the media	Informing about sports tourism
		Dissemination of the desired concepts and values of sports tourism
		Having specific and coherent policies and strategies in the direction of promoting sports tourism
		Introducing sports tour attractions
		Introducing jobs related to sports tourism
		Persuading the private sector to invest in sport tourism
		Showing enjoyable experiences from sports tours
		Awareness and clarification of issues and problems in
		development of sports tourism
	Natural attractions	Attractions related to winter sports
		Attractions related to summer sports
		Attractions related to water sports
		Attractions related to mountaineering and hill climbing
		Attractions related to nature tourism
	The role of the tour guide (Leader)	Having the necessary experience
		Knowing the principles of speech
		Having the necessary knowledge of the area
		Having great problem-solving skills
		First aid skills
		Having the necessary knowledge and awareness
		Knowing the English language
		Knowing the local language
		Having organizing and leadership skills
		Ability to communicate
	The quality of service	Access quality
		Accommodation quality
		Environmental health quality
		Development of communication ways and modern means of transportation
		Development of airline services
		Improvement of health facilities
		The quality of welfare and hospitality services
		Infrastructure quality
	Information Technology	Online booking of accommodation and hotel
		Using the capacity of social networks to advertise sports tours
		Considering virtual sports tourism as important
		Tourist database
		Virtual travel community
		Sharing tour content on the website
		Electronic visa development
	Support	Reducing taxes on flight tickets
		Lifting travel restrictions
		Paying part of the expenses
		Allocating the necessary budget for sports tours
		Formation of sports tourism federation
		Simplifying processes for tourism businesses
		Government support for sports tourism
	Culturalization	Encouraging people, organizations and Iranians abroad to invest in sports tourism
		Introducing the sports tourism capacities of Iran
		Interaction with cultures and civilizations of other nations
		Cultural capital
		Generalization of the culture of tourism development
		Holding traditional sports festivals
		Spreading the concepts and values of sports tourism
		Creating a systematic system for internal advertising of international tourists
	Education and training of human resources	Training of efficient human resources
		Training of the police guide
		Tourism education in the framework of responding to tourism demand
		Establishing qualitative guidance in education
		Adapting sports tourism curricula to environmental needs

#### 4.2.1. The role of the media

"Role of media" is a category due to which sports tourism undergoes a kind of positive evolution. This sub-category includes eight concepts, and one of the most frequent concepts is the introduction of sports tours. In this regard, interviewee number 13 stated: "The media has a strong role in the development of sports tours, because through the media, the attractions of sports tours are introduced to the people and domestic and foreign audiences, and through this, more tourists tend to participate in the relevant tours and as much as the media perform their duties in relation to tourism issues in the best way, parallel to that, the number of tourists in the country increases, because in this way, concepts and values of sports tourism will be introduced and the enjoyable experience of sports tours will be displayed."

#### 4.2.2. Natural attractions

Natural attractions which are derived from the five concepts of attractions related to winter sports, summer sports, water sports, climbing and hill climbing, and nature tourism, it indicates that sports tours take part of their transformation and development from natural attractions and as long as there are not enough attractions, it is not possible for tourists to be encouraged to participate in sports tours. Interviewee No. 4 stated: "No matter how much we invest in management and structural issues and have any number of plans and strategies, but if we do not have attraction and we cannot use these attractions, which sometimes some of them are man-made, we do not experience any development in practice, because attractions are part of the necessities of tourist destinations and tourists are basically encouraged because of this attraction.

#### 4.2.3. The role of the tour guide (leader)

According to the participants, along with natural attractions, tour guides are one of the elements that play a prominent role in the development of sports tours and the sports tourism industry, since guides are at the forefront of service delivery and communicate directly with tourists without any intermediaries, and their behavior and actions strongly influence tourists. In this context, the following two quotes are noteworthy. First, participant number 12 stated: "The development of sports tours, in general, sports tourism is subject to various factors, but one of the human factors that plays the most important role in the field of development is the tour guides, who must have the necessary and sufficient experience and be fluent in the international language and the local language as well."

Interviewee No. 15 also stated: "A tour guide is an influential person on the development of sports tours and has a prominent and un-negligible role in the development, so this person must have sufficient knowledge of the region and must be very capable and expert in communicating with tourists".

#### 4.2.4. The quality of service

Another facilitator that has been proven to be effective on tourism and its development since ancient times is the category of service quality. The informants who were studied identify quality as one of the most important key drivers of development, and according to them, quality is one of the obligations of development, and without it, even if all other drivers of development are available, the development of the form does not take, as the development is both affected by the quality of services and affects it, and they are somehow necessary for each other.

#### 4.2.5. Information technology

The introduction of information technology into the lives of people on the planet, especially sports tourism, is a very important issue. Because we are currently living in the digital age and we are witnessing rapid and exponential changes every day, and sports tourism has undergone changes accordingly, and by using this technology, we can create a huge transformation in tourism. With the help of tourism technology, it has grown significantly in the past years. In this regard, interviewee number 1 stated: "Today, it is no longer possible to provide services in a traditional way and it is not possible to act based on the traditional actions of the past." In order to witness the development and transformation in tourism, we must consider information technology as important and emphasize on the development of electronic visa and virtual travel community."

#### 4.2.6. Support

Another issue that has been proven many times, like the quality of service, has revolutionized sports tourism and led it towards development, is support. The support itself is derived from the seven concepts of reducing taxes on flight tickets, removing travel restrictions, paying part of the costs, allocating the necessary budget for sports tours, forming a sports tourism federation, simplifying processes for tourism businesses and the government supports for tourism.

#### 4.2.7. Culturalization

Another extracted sub-category is "culturalization". The meaning of this category is that as a result of cultural development, the development of the sports tourism industry and sports tours is possible. Because sports tourism and tourism in general is a socio-cultural phenomenon that is both influenced by culture and influence it. No matter how much cultural development is pursued with seriousness and planning, it is equally likely that sports tours and the tourism industry will be transformed. In this regard, interviewee number 10 stated: "We should look at culturalization as a long-term strategy for development and in order to be able to attract foreign investors, and to establish optimal interaction with other cultures of the world and to encourage tourists to travel to the country, we must develop culture internally and improve our cultural capitals."

#### 4.2.8. Education and training of human resources

The last facilitator and accelerator that was extracted in this research is "human resources education and training". According to this category, one of the determinants of the development of sports tourism in Iran is to train suitable human resources in the field of sports tourism and sports tours. This sub-category is derived from the five concepts of training efficient human resources, training guide police, tourism training in the framework of responding to tourism demand, creating quality guidance in education and adapting sports tourism curricula to environmental needs.

The above content was dedicated to the story line based on the grounded theory regarding the development of sports tours in Iran’s tourism industry. The topics presented above have been reconstructed based on the semantic process of development limiters and facilitators. This story line showed that the development of sports tours in Iran is influenced by what conditions and also depicted the role of facilitators and limiters.

## 5. Discussion

The purpose of this research was to present the development model of sports tours in the tourism industry. The findings of this research showed that the development of sports tours is subject to two main categories, one of which plays the role of facilitator and the other plays the role of the limiter. Among these, the role of the media, natural attractions, the role of the tour guide, service quality, information technology, support, culture building, education, and training of human resources are among the facilitating factors and financial, political and security, structural and organizational issues. Were among the limiting factors.

This finding relatively satisfies the results of the studies of Heydari et al. [18] in which they showed that technology, inflation and wear and tear, and lack of responsiveness of infrastructure are the most important macro environmental limiters to the development of sports tourism. Also, Pezhvakmoghadm [15] had come to the conclusion that there is a significant relationship between the Internet and social networks and the reuse of services and advertisements regarding sports tourism tours; Further, Keivanihafshejani et al. [16] showed that governmental and legal obstacles have the highest impact on the lack of development of sports tourism; Abedi Samakosh et al. [17] pointed to natural attractions; Shahriary et al [12] revealed that structural unity is effective on development; Yang et al. [14] mentioned the governmental support; and Jiménez-García et al. [13] emphasized that education as an effective factor. No inconsistency was found in the literature.

The reason that the media helps in the development of sports tours is that the media plays the main role in the development of the tourism industry and the introduction of tourist attractions, and in this way, they can be well suitable for the implementation and completion of tourism projects. Today, the most important task of the media is to conceptualize and create mental images of facts and even non-facts; The media play an important role in presenting real and unreal images of regions due to their broad capabilities in attracting a mass audience and the ability to shape public opinion, and they are very important for people in depicting different places and countries and shaping geographical ideas. Moreover, the reason why natural attractions have a facilitating role is that knowing the capabilities of tourism with the focus on creating attractions is the first important step in the development of sports tourism. In fact, the success of sports tourism is completely dependent on the capabilities of tourist destinations, which are unique and distinct compared to other destinations. Also, the reason why the role of the tour guide was recognized as a facilitating factor is that the tour guide is in the first line of providing services, on the other hand, tourists are in contact with the tour guides most of the time. As a result, his/her behavior and reaction in different situations definitely have an effect on tourists. However, a tour guide can sometimes help in the development of sports tours that benefit from special and noble personalities and behavior, so that they can survive.

The reason that was recognized that the quality of service helps the development of sports tours is that the quality of service is the main core of tourism marketing with a major emphasis on various fields of tourism. In recent years, paying attention to the needs of tourists and responding to their demands in the service sector has been one of the main and essential tasks or goals of sports tourism development. This is despite the fact that service quality has a long history and has been identified and emphasized in the first research in the field of tourism. However, it can be seen that despite the passage of a long time since the issue of service and its measurement and evaluation methods, not only attention to this issue has not decreased during the last decade, but it has become more important because of its increasing importance among tourists. On the other hand, the quality level of sports tourism services determines the amount of tourist attraction. In other words, as long as the quality of the services provided in the form of natural and unnatural landscapes is at a favorable level, it will create satisfaction and ease of mind for the visitors. Accordingly, in return, improving the level of satisfaction with the services provided will also increase the number of tourists, which can be the basis for development. Because a satisfied tourist is a source of profit, and areas that can get it to increase their profits.

In addition, the reason that information technology was recognized as a facilitator is that tourism is an information-intensive industry. Tourists bear considerable risk by spending money and time on travel. The higher the risk, the more experiences it will bring to the passengers. The tourism business is a good opportunity to minimize the percentage of risk, and the expansion of online technology makes consumers more options to choose from and get the information they need from trusted sources, which are considered essential elements of tourism. Since consumers seek to gain awareness and collect data from various sources, sports tourism businesses should invest in creating a comprehensive and informative sustainable strategy. In addition, the reason for choosing support as a contributing factor was that the development of tourism destinations requires effective and all-round support from the people of the organizations in charge, especially the government, to accompany and align the stakeholders of tourism and expand the satisfactory interactions of tourists and host communities. As a result of these constructive and effective interactions, tourism benefits for tourists and host communities are enhanced and can provide the grounds for national development.

In addition, the reason that culture was among the facilitating factors is that society is a kind of social system. The social system is the result of social relations, which, unlike natural relations, are not definite and developmental matters. Certainty does not prevail in human systems due to the presence of agency and awareness, and in certain circumstances, humans can give different answers to a specific factor, and these multiple answers are nothing but the existence of plurality and different options. It is with the multiplicity and existence of different options that a phenomenon called (choice) becomes meaningful. Different choices are made based on different mental values of people, which is actually culture that shapes different values ​​for different people and societies. Therefore, it can be seen that there is a close relationship between tourism and culture.

Finally, the reason that it was recognized that the education and training of human resources help in the development of sports tours is the formation, sustainability, and development of tourism in different types in this research on sports tourism and among that sports tours in every society requires an educational. The understanding and knowledge of tourism as well as how to develop and sustain it lies not only in expanding the knowledge of tourism to understand its needs but also in teaching the areas of success in the tourism business and also the needs of each society towards tourism, which is an attitude in itself, which makes a human approach to tourism necessary. On the other hand, tourism education is placed on both sides of a process of tourism knowledge processing, which, on the one hand, has played a role in explaining the knowledge of tourism, and on the other hand, reveals how tourism works in the land and other lands. Meanwhile, tourism education is an approach to providing the necessary human resources for the development of tourism. On this basis, tourism education works in the framework of responding to the demand of tourism in terms of the required human sources, which is in accordance with the current and prospective real needs.

## 6. Strengths and limitations

In this study, since the data theory method of the Glaser’s approach was used, after the analysis of the data from the interview, the related literature was reviewed, and before that, the research literature was not reviewed. For this reason, grounded theory methodology was adopted to develop the theory of sports tour development. The accurate use of the data theory methodology of the Glaser’s approach guarantees the authenticity, validity and usefulness of the theory of the development of sports tours based on facilitators and limiters of development. This study included participants from all levels and positions who worked in various fields related to tourism and sports tours with different genders and educational backgrounds, which has ensured diversity in the sample. This theory can be used in the context of Iran and can also be generalized in other countries with similar socio-cultural characteristics, especially West Asian countries. The main limitation of this study is the selection of some participants from government organizations based on their strategic positions, which may not have mentioned all the limitations and obstacles due to their position in the relevant organization. In this study, key informants were interviewed, and the opinions of the ordinary people, especially tourists, were not reflected, and there may be differences between their opinions. Another limitation of this research is climate change, which can be one of the effective limiters, however, in this research, there was no specific index for statistics.

## 7. Conclusion

Adapting from what was said, it seems that there are various drivers in the development of sports tours, some of which are facilitating and some are limiting. Based on this result, it is suggested to the policymakers and Decision-Making Bodies related to sports tourism and sports tours to minimize the existing obstacles and remove them if possible, and on the other hand, strengthen facilitating factors that help the development of sports tours.

## Supporting information

S1 File (DOCX)
