# Supplementary material for: Providing a model for the development of sports tours in the tourism industry
Source: PLoS One. 2023 May 25;18(5):e0285457. doi: 10.1371/journal.pone.0285457 (PMC10212106; doi:10.1371/journal.pone.0285457)
Supplement: S1 File — (DOCX) [file pone.0285457.s001.docx]

**Dear PLOS ONE editorial team,**

We appreciate your efforts to promote our scientific research and would like to provide the following descriptions of the research, which we hope will be helpful.

1. The article presents data from demographic research. This information is given in this segment.

**Table 1**- Demographic characteristics of the participants

| **Age (years)** | **Field of study** | **Education** | **Experience (Years)** | **Position/area of activity** | **Row** |
| --- | --- | --- | --- | --- | --- |
| 29 | Political geography | Ph.D. student | 5 | The employee of the ministry of cultural heritage and tourism | P1 |
| 49 | Tourism management | Masters | 26 | Managing director of nature tourism company | P2 |
| 47 | Sport Management | Masters | 9 | Chairman of the sports tourism committee of the ministry of cultural heritage and tourism | P3 |
| 45 | Tourism management | Ph.D. student | 20 | Head of the codification office of the ministry of cultural heritage and tourism | P4 |
| 49 | Tourism management | Ph.D. | 15 | Head of the general supervision department of the ministry of cultural heritage and tourism | P5 |
| 43 | Geography and tourism planning | Ph.D. | 21 | Editorial board of the quarterly journal of tourism research and sustainable development | P6 |
| 40 | Business Management | Ph.D. | 18 | Business consultant in tourism start-ups | P7 |
| 44 | Business in Tourism | Ph.D. | 17 | Managing director of travel and tourism services company | P8 |
| 42 | Ancient languages of Iran | Masters | 17 | Managing director of travel and tourism services company | P9 |
| 37 | Sport Management | Masters | 6 | Business consultant in sports tourism startups | P10 |
| 31 | Sport Management | Ph.D. student | 8 | Managing director of a non-governmental organization active in the field of sports tourism | P11 |
| 48 | Tourism management | Masters | 17 | Sports tour guide | P12 |
| 45 | Tourism management | Ph.D. student | 11 | Managing director of nature tourism company | P13 |
| 35 | Business Management | Masters | 8 | Sports tour guide | P14 |
| 37 | Tourism management | Ph.D. student | 13 | The employee of the sports tourism committee of the ministry of cultural heritage and tourism | P15 |

1. The interview files that were recorded as the voice of the interviewees in the research were the research's main data, or meta-data. These sound documents incorporate a totally open meeting and two-way collaboration of the principal creator of the exploration as a questioner (Mahdi Gharibzadeh) and different interviewees. The interviewees were fully aware of how to record and use the equipment when the files were collected. The interviewees gave their full consent, and they were able to express their thoughts and strategies in Persian, which was a clear language. These interviews began with the writers' team asking predetermined questions and continued as open discussions until the final summary was produced. These documents were utilized for nitty gritty handling and investigation.
2. It should be recalled that the interview file with the experts serves as the information source for the current study. The interview information has only been analyzed using the coding method, and no quantitative statistical procedures have been carried out on it. In this regard, the codes were chosen manually using Maxqda, NVIOV, and other software. we’re not utilized. It is important to note that the method used to code the interview data is outlined in the table below. Additionally, the magazine will receive the interview audio recordings.

**Table 2-** Sample open coding

| **Open code** | **Interview text** | **Indicator** |
| --- | --- | --- |
| Spreading the desired concepts and values of sports tourism | In my opinion, one of the most important factors that play a role in explaining the growth and optimization of sports tours is to expand the concepts and values of sports tourism in the first step. | P1 |
| Introducing sports tour attractions | Another thing is that it is necessary to introduce the attractions of sports tours to the audience of these tours and help them to know them better. |  |
| Attractions related to winter sports - attractions related to water sports | It is also necessary to introduce the attractions of sports tours, the trustees or the people who are responsible in this field should introduce other attractions, including winter and water sports attractions because this attraction is based on the theory of action in the behavior of tourists. It has a direct effect and, in some way, encourages it to make a tour. |  |
| Having the necessary experience - knowing the local language - knowing the English language | Another thing that is very important is that our tour leaders have enough experience. If this is not the case, no one would be willing to participate in a sports tour. Compared to the experience of tour leaders of other tours, more than The experience of tour leaders is sports, which is a big gap, besides that, a professional tour leader needs to know English and even the local language, this is one of the basic characteristics of a professional tour leader. |  |

**Table 3-** Concepts, sub-categories, and main categories affecting the development of sports tours

| **Indicator** | **Open codes** | **Axial code** | **Theoretical code** |
| --- | --- | --- | --- |
| P2, P4, P6, P11 | Informing about sports tourism | The role of the media | The Facilitators |
| P1, P2, P8, P9, P12 | Dissemination of the desired concepts and values of sports tourism |  |  |
| P2, P3, P11, P14 | Having specific and coherent policies and strategies in the direction of promoting sports tourism |  |  |
| P1, P3, P4, P6, P8, P9, P10, P12, P13 | Introducing sports tour attractions |  |  |
| P9, P11 | Introducing jobs related to sports tourism |  |  |
| P9, P11, P13, P14 | Persuading the private sector to invest in sport tourism |  |  |
| P4, P8, P9, P11 | Showing enjoyable experiences from sports tours |  |  |
| P5, P4, P6, P10 | Awareness and clarification of issues and problems in |  |  |
|  | development of sports tourism |  |  |
| P1, P2, P3, P4, P6, P8, P9, P11, P12, P13, P14 | Attractions related to winter sports | Natural attractions |  |
| P4, P5, P8, P12, P14 | Attractions related to summer sports |  |  |
| P1, P2, P4, P5, P6, P8, P9, P11, P12, P13 | Attractions related to water sports |  |  |
| P2, P3, P4, P6, P7, P11 | Attractions related to mountaineering and hill climbing |  |  |
| P7, P13 | Attractions related to nature tourism |  |  |
| P1, P2, P4, P5, P6, P7, P8, P9, P11, P12, P13 | Having the necessary experience | The role of the tour guide (Leader) |  |
| P2, P3, P5, P6, P8, P11 | Knowing the principles of speech |  |  |
| P6, P7 | Having the necessary knowledge of the area |  |  |
| P6 | Having great problem-solving skills |  |  |
| P6, P9, 12 | First aid skills |  |  |
| P2, P3, P5, P6, P9, P12, P14 | Having the necessary knowledge and awareness |  |  |
| P1, P2, P4, P5, P6 | Knowing the English language |  |  |
| P1, P4, P6 | Knowing the local language |  |  |
| P6 | Having organizing and leadership skills |  |  |
| P6, P13, P15 | Ability to communicate |  |  |
| P1, P2, P3, P5, P6, P8, P10, P11, P12, P13, P14 | Access quality | The quality of service |  |
| P1, P2, P4, P5, P6, P7, P8, P11 | Accommodation quality |  |  |
| P3, P5, P8, P9, P11 | Environmental health quality |  |  |
| P9, P11, P12 | Development of communication ways and modern means of transportation |  |  |
| P2, P4, P5, P8, P11, P12, P15 | Development of airline services |  |  |
| P1, P2, P4, P5, P6, P7, P9, P11, P14, P15 | Improvement of health facilities |  |  |
| P3, P4, P5, P6 | The quality of welfare and hospitality services |  |  |
| P2, P3, P4, P6, P8 | Infrastructure quality |  |  |
| P3, P4, P5, P7, P8, P10 | Online booking of accommodation and hotel | Information Technology |  |
| P2, P3, P8 | Using the capacity of social networks to advertise sports tours |  |  |
| P2 | Considering virtual sports tourism as important |  |  |
| P2, P11, P12 | Tourist database |  |  |
| P2 | Virtual travel community |  |  |
| P2 | Sharing tour content on the website |  |  |
| P2, P4, P11, P13 | Electronic visa development |  |  |
| P3, P4, P11, P14 | Reducing taxes on flight tickets | Support |  |
| P3, P5, P6, P8, P10, P11 | Lifting travel restrictions |  |  |
| P2, P5, P8 | Paying part of the expenses |  |  |
| P1, P2, P6, P7, P8, P9, P11 | Allocating the necessary budget for sports tours |  |  |
| P2 | Formation of sports tourism federation |  |  |
| P1, P3, P5, P6, P7, P8, P9, P11, P13 | Simplifying processes for tourism businesses |  |  |
| P1, P2, P3, P5, P6, P7, P8, P11, P13 | Government support for sports tourism |  |  |
| P3, P5, P6, P8 | Encouraging people, organizations and Iranians abroad to invest in sports tourism | Culture building |  |
| P2, P4, P5, P6, P7, P11, P14 | Introducing the sports tourism capacities of Iran |  |  |
| P2, P4, P5 | Interaction with cultures and civilizations of other nations |  |  |
| P2 | Cultural capital |  |  |
| P3, P4, P8, P11, P12 | Generalization of the culture of tourism development |  |  |
| P2, P8 | Holding traditional sports festivals |  |  |
| P9, P11, P13 | Spreading the concepts and values of sports tourism |  |  |
| P5 | Creating a systematic system for internal advertising of international tourists |  |  |
| P2, P3, P4, P6, P7, P8, P9, P11, P14 | Training of efficient human resources | Education and training of human resources |  |
| P9 | Training of the police guide |  |  |
| P2, P4, P9, P14 | Tourism education in the framework of responding to tourism demand |  |  |
| P9 | Establishing qualitative guidance in education |  |  |
| P9, P15 | Adapting sports tourism curricula to environmental needs |  |  |
| P1, P4, P6, P8, P10 | People's purchasing power decline | Financial issues | The limiters |
| P2, P5 | The high cost of tours |  |  |
| P2 | The variable rate of tours |  |  |
| P2, P4, P8, P11 | Lack of interdepartmental/organizational coordination and integration | Structural issues |  |
| P3, P4, P6, P7 | Interference of duties of organizations related to tourism |  |  |
| P8, P4 | The multiplicity of decision-making centers |  |  |
| P6, P8, P11, P12, P13, P14, P15 | Lack of incentive policies for private sector participation | Organizational issues |  |
| P3, P5, P7 | Absence of comprehensive sports tourism development plan |  |  |
| P8 | Non-specialized management of sports tourism |  |  |
| P8, P12 | Short-term policies in the field of tourism |  |  |
| P3, P5, P8 | Lack of belief in sports tourism | Political and security issues |  |
| P8, P9, P11 | International relations |  |  |
| P8, P15 | Dependence on oil |  |  |
| P6, P7, P11 | Diplomatic problems |  |  |
| P8 | Negative propaganda against Iran |  |  |
| P8, P13 | Domestic incidents against international tourists |  |  |
| P8, 12, P13 | Embargo |  |  |

1. The DOI provided in the attachment provides access to the interview's audio files:

Figshare DOI: 10.6084/m9.figshare.22783025
